# The effect of Phytopaj *)Ferula assa-foetida* L. oleo gum resin and tragacanth( in patients with COVID-19: A randomized clinical trial

**DOI:** 10.22038/AJP.2023.22800

**Published:** 2024

**Authors:** Hamid Reza Bahrami-Taghanaki, Hamidreza Hoseinzadeh, Shokouhsadat Hamedi, Majid Jafari Nejad-Bajestani, Nayereh Esmaeilzadeh, Hasan Abdollahzadeh, Seyedehmasoume Hoseini-asil, Gholamreza Haghighi, Amin Bojdi

**Affiliations:** 1 *Department of Complementary and Chinese Medicine, Faculty of Persian and Complementary Medicine, Mashhad University of Medical Sciences, Mashhad, Iran *; 2 *Department of Persian Medicine, School of Persian and Complementary Medicine, Mashhad University of Medical Sciences, Mashhad, Iran*; 3 *Department of Clinical Persian Pharmacy, School of Persian and Complementary Medicine, Mashhad University of Medical Sciences, Mashhad, Iran*; 4 *Department of Epidemiology, School of Health, Mashhad University of Medical Sciences, Mashhad, Iran*; 5 *Department of Infectious Diseases and Tropical Medicine, * *Faculty of Medicine* *, Mashhad University of Medical Sciences, Mashhad, Iran*; 6 *Department of Traditional Medicine, School of Medicine, Zabol University of Medical Sciences, Zabol, Iran*

**Keywords:** COVID-19, Herbal medicine, Integrative medicine, Persian medicine, Randomized controlled trial

## Abstract

**Objective:**

Exogenous hydrogen sulfide (H_2_S) has a positive effect on respiratory diseases. Oleo-gum of *Ferula assa-foetida* contains this compound. This study assessed the effects of *Ferula assa-foetida* L. oleo gum resin and tragacanth (Phytopaj) on patients with COVID-19.

**Materials and Methods:**

A randomized, single-blinded, controlled trial (RCT) phase 2 was conducted in Mashhad on hospitalized COVID-19 patients. In this RCT, 122 patients were randomly assigned to either receive a 14-day oral phytopaj plus ordinary treatment or ordinary treatment only. Changes in peripheral blood lymphocyte count (LC) and blood oxygen saturation (PO_2_) were the endpoints.

**Results:**

Mean±SD of PO_2_ in Phytopaj comparison ordinary treatment before intervention was 91.86±4.62 and 91.41±9.18, after the intervention it was 93.22±4.26 and 91.91±5.92 mmHg; before intervention, mean±SD of peripheral blood lymphocyte count was 1015.90±500.55, and 1104.28±543.61, and after intervention, it was 1652.27±921.38 and 1326.12±719.28/μL respectively.

**Conclusion:**

Phyopaj is most useful in moderate stages of Covid19, and it is not recommended for elderly patients and patients with comorbidity until more insight is gained.

## Introduction

Globally, Coronavirus disease 2019 (COVID-19) has emerged as a pandemic with high hospitalizations and morbidities, making the healthcare system unable to provide effective and accessible treatments (Wang et al., 2020; Esmaeilzadeh et al., 2023).

SARS-CoV-2 infection can cause a variety of signs and symptoms. In patients with mild to moderate COVID-19 illness, mild fever, chills, dry cough, sore throat, headache, nasal congestion, and myalgia are common symptoms. Diarrhea and vomiting are some of the symptoms of gastrointestinal infection found in some patients. ARDS (Acute Respiratory Distress Syndrome) and death are the results of complicated COVID-19 forms caused by massive lung damage (Jiang et al., 2020). As of yet, there is no definitive treatment for COVID-19 infection, but a number of medicines have been proposed, including lopinavir/ritonavir, remdesivir, imatinib, tocilizumab, infliximab, and artesunate (Lamontagne et al., 2020; Nicola et al., 2020).

There has always been a major role for herbal medicine in treating various illnesses throughout history (Abdollahzadeh et al., 2021; Muhammad, 2020), and people prefer plant-based drugs over chemical medicines, particularly in developing countries (Mahendra and Bisht, 2020; Ameri et al., 2015). Since only one-quarter of drugs are derived from plants, the value of herbal medicines has yet to be fully appreciated (Kim, 2005).

Persian medicine (PM) has a history in Iran dating back more than ten centuries (Iranzadasl et al., 2020). The abundance of literature on herbal plants' use as treatments for a variety of diseases, including respiratory diseases, makes it possible to identify novel antiviral drugs against COVID-19 using PM and natural products, which can make treatment regimens accessible and affordable. 

 There are more than 170 species of *Ferula* in central Asia (eastern Iran, west Afghanistan, Iraq, and Turkey), Europe, and North Africa.  *Ferula assa-foetida* which is more common in Iran and Afghanistan, has a sweeter flavor and is bitterer (Amalraj and Gopi, 2017; Sahebkar and Iranshahi, 2010). In Persian, *Ferula* gum is called *'Anghuzeh', 'Haltit'* or *'Tyib'* in Arabic, *'Hing'* in *Hindi*, '*Mvuje'* in Swahili, *'Asafetida'* in Spanish, *'Awei'* in Chinese, *'Merde doubtable'* in French, and *'Stinkasant'* or '*Teufelsdreck*' in Germanic languages (Mahendra and Bisht, 2020). 

Various disorders can be treated with *Ferula assa-foetida* (Mahendra and Bisht, 2020; Dehpour et al., 2009; Kavoosi and Rowshan, 2013; Shrivastava, 2012; Divya et al., 2014; Kamble and Patil, 2008; Saleem and Sultana, 2001; Abu-Zaiton, 2010; Fatehi, 2004; Bagheri et al., 2014; Moghaddam and Farhadi, 2015; Kumar and Singh, 2006). In addition, it is used to treat whooping cough, asthma, and chronic bronchitis as an expectorant (Karimian et al., 2021). A recent study showed that the oleo-gum resin of *Ferula assa-foetida* significantly improved clinical presentations of COVID-19 including cough, dyspnea, myalgia, anorexia, anosmia, and sense of taste (Hasanpour et al., 2022). 

the *Ferula assa-foetida* gum contains a water-soluble part of oligosaccharides that typically exhibit no pharmacological activity (Srinivasan, 2005; Khalilova et al., 2013; king et al., 2014)

Several biological functions are modulated by hydrogen sulfide (H_2_S), and its depletion contributes to illnesses. A physiological level of H_2_S reduces inflammation throughout the body and scavenges peroxonitrite and reactive oxygen species (ROS) (Calderone et al., 2016). H_2_S, both endogenous and exogenous, enhances respiratory function by modulating mucolytic activity and decreasing mucus viscosity (Bazhanov et al., 2017). Mucin breakage is caused by disulfide bond interaction (Costantin et al., 2006). As a result of H_2_S 's increased expression of NOS and NO bioavailability, the airways are indirectly protected against viral infections (King et al., 2014). Exogenous H_2_S has been investigated in recent decades as a treatment for respiratory diseases (Citi et al., 2020). According to a document, H_2_S-releasing compounds have antiviral properties as well as improve immune defenses against viruses (Wang et al., 2017). 

Tragacanth is another herbaceous plant. Iran and Turkey have dry mountainous regions where it grows (Tarkesh, and Jetschke, 2016). The other names of this plant are ‘*Adragante’, ‘Astragale Adragant’, ‘Astragale de Marseille’, ‘Astragalus gummifer’, ‘Coussin-de-Belle-Megravere’, ‘Goat’s Thorn’, ‘Gomme Adragante’, ‘Gomme de Dragon’, ‘Green Dragon’, ‘Gum Dragon’, ‘Gummi Tragacanthae’, ‘Gum Tragacanth, Hog Gum’, ‘Syrian Tragacanth’, ‘Tragacanth Gum’, ‘Tragacanthe’*, and ‘*Tragacanto’* (Yakuboğulları, 2019). Tragacanth is traditionally used with *Ferula assa-foetida* as a laxative and reduces its negative effects (Aghili, 2009).

In addition to being antiviral*, Ferula assa-foetida* gum and tragacanth are easy to access, accepted in the community, and are cost-effective options for treating COVID-19. However, they must be demonstrated to be effective in treating COVID-19 by scientific evidence-based studies. Therefore, we conducted this study taking into consideration the healing properties of *Ferula assa-foetida* and tragacanth (Phytopaj) and the urgent need to find affordable and accessible COVID-19 remedies.

## Materials and Methods

**Preparation of medications**

Plant collection: *Ferula assa-foetida* and tragacanth gum were prepared from a standard herbal shop in Mashhad, Iran. The herbarium voucher number was determined by the botanist of the Department of Botany Research at the Department of Pharmacognosy, school of pharmacy, Mashhad University of Medical Sciences, Mashhad, Khorasan Razavi, Iran (voucher Number of *Ferula assa-foetida*: 293-0606-2, voucher number of tragacanth: 775-1). The gums were dried in the shade and completely ground with a mill separately.  

**Preparation of herbal capsules**

A 1:2 ratio was used to combine the sifted ground powder of each gum. This combination was added to the resulting mixture of avicel powder and baking soda as fillers (flowable and lubricant). This powder has a spicy taste with a strong sulfur odor, and by means of the manual capsule filling machine, the capsules were filled with the powder mixture. Capsules contained 300 mg of soft *Ferula assa-foetida* gum and 125 mg of soft tragacanth gum, according to Mahendra and Bisht, 2012; Goudah et al., 2015; Lee et al., 2009; Ghannadi et al., 2014). 

**Effective substance of medication**

Terpenoids and sesquiterpenes are effective constituents of *Ferula assa-foetida gum resin. *The metabolites such as *β-Pinene, α-Pinene, Propyl n-butyl* *disulfide*, and 1, 2*-dithiolane, (Z)-1-propenyl sec-butyl disulfide*, and *β-Eudesmol* are the main constituents of the essential oil of this gum (Bahrami et al., 2020). 

This is in vitro evidence that polysaccharides of tragacanthin can activate peritoneal macrophages and provide protection for animals against infection (Abas et al., 2014). 

**Standardization of herbal capsules**

The herbal capsules were standardized, based on their total phenol, using the Folin–Ciocalteu method (Hamedi et al., 2016). Mixed powder was evaluated as a standard for total phenolic content using gallic acid (GA). The *Folin–Ciocalteu* method was applied to determine total phenolics calorimetrically. Total phenolics were determined using a calibration curve obtained from measuring the absorbance of a known concentration of GA standard. The concentrations are expressed as milligrams of GA equivalents per capsule (Amini et al., 2019). The amount of tannin equivalent to GA was 224 µg per capsule (Amini et al., 2019). 

**Study design**

**Clinical trial site**

 This is a parallel 2-arm randomized, controlled, single-blinded study to evaluate the effect of *Ferula* *assa-foetida *L.* oleo* gum resin and tragacanth in hospitalized patients with confirmed COVID-19 based on a positive Polymerase Chain Reaction (PCR*)* laboratory test or radiographic manifestations on lung CT scan. This study was conducted at Imam Reza hospital, Mashhad, Iran. This trial protocol was approved by the Ethics Committee of Mashhad University of Medical Sciences (Ethics ID: IR.MUMS.REC.1399.285), and it was registered in the Iranian Registry of Clinical Trials with Registration (No. IRCT20200607047675N1).

 There were no major errors in this study's design, and it met all 20 items listed in the International Clinical Trials Registry Platform (ICTRP) for registration as well as ICTRP's 20 criteria for eligibility (World Health Organization, 2020b). According to CONSORT (Consolidated Standards of Reporting Trials) and Standard Protocol Items for Randomized Trials guidelines, the protocol of the study was strictly followed (Bian et al., 2011).

An informed consent form was signed by all patients or their legal representatives prior to participating in the trial. In addition, demographic data and medical histories were collected from patients.

**Inclusion criteria**

The study included hospitalized patients at Imam Reza hospital (Mashhad, Iran) from July 28 to September 1, 2020, who were recently diagnosed with COVID-19 based on a positive PCR laboratory test or diagnostic chest CT scan and were aged 18 to 75 years old. According to the eighth edition of the national guidelines for treating COVID-19, they were in moderate to severe stages and agreed to participate voluntarily.

**Exclusion criteria**

Patients in critical stage, end-stage heart failure, recent cardiac intervention less than 2 months (coronary angioplasty, Implantable cardioverter defibrillator (ICD), coronary artery bypass graft (CABG), valvuloplasty or replacement), pulmonary fibrosis or advanced chronic obstructive pulmonary disease (COPD), end-stage kidney disease or liver disease, active tuberculosis or active hepatitis, or undergoing immunosuppressive treatment, pregnancy or lactation, or sensitive to herbal medicine, were exclusion. 

**Randomization and masking**

A SPSS randomization plan was used to assign patients to Phytopaj/ ordinary treatment or ordinary treatment only in a 1:1 ratio. The sealed envelope method was used for allocation concealment. Both the physicians and patients were aware of the allocation of treatments. The physicians who assessed the interventions' outcomes and the statistician who analyzed the data, however, were blinded to the study allocation.

**Sample size determination**

For sample size calculation, the result of a study showed that patients with SARS treated with integrated treatment had an increase in peripheral blood lymphocytes (0.98 x 0.65 x 109/l) compared to conventional treatment alone (0.59 x 0.34 x 109/l). The samples for each arm were 61 according to “n=2[(Z_α/2 _+ Z_β_)^2^]/ (µ_1_ - µ_2_) ^2^ equation; where n = sample size required in each group, µ_1_= mean of subject cured by integrated treatment, µ_2_= mean of subject cured by conventional medicine only, µ_1_ - µ_2_=clinically significant difference, Z_α/2_: This depends on level of significance, for 5% this is 1.96, two tail, Z_β_: This depends on power, for 80% this is 0.84    

 A total of 140 patients were screened to determine their eligibility for the study. It was found that 122 patients had met the study's screening criteria.

**Intervention**

In the trial, the patients were assigned randomly to one of two arms: one arm consisted of phytopaj administered twice daily by mouth after breakfast and dinner for 14 days, along with an ordinary treatment, and the other arm consisted of an ordinary treatment alone.

Using the national guidelines for treating COVID-19 in Iran, this reference manual is used to treat COVID-19 (Lopinavir/Ritonavir, Interferon beta-1b, Enoxaparin and dexamethasone). To ensure good participant adherence, direct observation was applied to the admission of medicines.

**Measurements**

 Case Report Forms (CRFs) were used to record demographic data after enrolling patients in the study. On day 1, both groups were evaluated for oxygen saturation (PO2), peripheral blood lymphocytes count (LC), and chest CT results. On the 14th day of the study, patients were examined regarding their PO_2_ level and LC. During the baseline and follow-up sessions, an infectious disease specialist and a nurse recorded these data. Pulse oximeters were used for measuring PO_2_ saturation, CTs were interpreted by a radiologist, and fresh blood samples were analyzed for peripheral blood lymphocyte count.

**Objectives and outcome measure**

A comparison was made between the two groups based on the difference in peripheral blood lymphocytes count and the degree of PO_2_. Due to the study design consisting of assessor judgment and patient reports, these outcomes reduced the risk of biases.

**Active monitoring ADR (adverse drug reaction)**

Adverse events such as fever, dermal disorders, renal failure, and gastrointestinal disorders were most commonly detected through active monitoring. After a period of time, three patients with altered dermis and gastrointestinal systems appeared in the phytopaj and ordinary treatment groups (lost to follow-up).

**Statistical** **analyses**

It appears that age, gender, comorbidities, and severity of Covid -19 can influence treatment outcome, so we evaluated their effect. Mean±SD was used to describe quantitative data. The student's t-test was used for between and within groups to compare continuous outcomes, and chi-square test was used for qualitative data. Throughout the paper, all p values and 95% CIs are reported as two-sided test results. All analyses were done with Stata14 software (StataCorp, College Station, TX, USA). 

## Results

**Participants and baseline characteristics**

**Figure 1:**
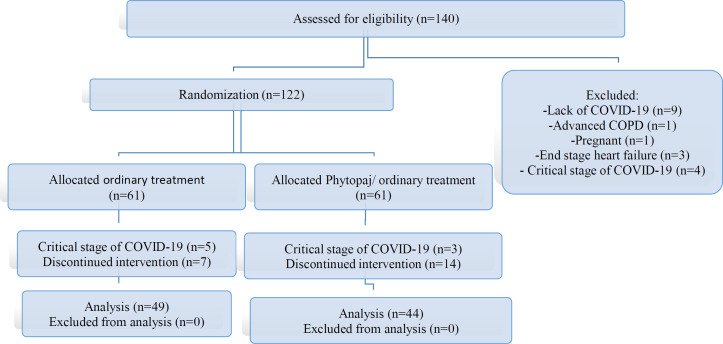
Study flow chart

Between July 28, 2020, and September 1, 2020, 140 patients were enrolled and evaluated for eligibility. A total of 122 out of 140 patients were eligible for the study and were randomly assigned to Phyopaj / ordinary treatment or ordinary treatment. In Phytopaj / ordinary treatment group, participants received a 14-day Phytopaj / ordinary treatment therapy, and in the other group, they received only the ordinary treatment. During the 14-day follow-up period, 14 and 7 participants failed to follow the study protocols in the Phytopaj / ordinary treatment and the ordinary treatment, respectively. As a result, they were excluded from the analysis, and 93 patients (76%) were analyzed in total (Figure1).The baseline characteristics of participants before receiving interventions are shown in Table 1. Gender, comorbidity, and disease severity did not show statistically significant differences between the two groups. However, the patients in the Phytopaj / ordinary treatment group were younger than those in the ordinary treatment group due to the refusal of elderly people to continue Phytopaj / ordinary treatment (the mean age of those not adhering to Phytopaj / ordinary treatment (12 patients) was 69 year). 

**Table 1:** Baseline characteristics of the study participants

Characteristic	All (93 patients)	Ordinary treatment (49 patients)	Phytopaj / ordinary treatment (44 patients)	p value
Age(year)	57.2 (15.5) N (%)	52.7 (14.0) N (%)	61.2 (15.7) N (%)	0.04^**^
Male	60 (64.5)	33 (55.0)	27 (45)	0.66^*^
Female	33 (35.5)	16 (52.0)	17 (48.0)	
With comorbidity	46 (49.5)	29 (63.0)	17 (37.0)	0.08^*^
Without comorbidity	47 (50.5)	20 (42.6)	27 (57.4)	
Moderate stage of COVID 19	65 (69.9)	37 (56.9)	28 (43.1)	0.26^*^
Severe stage of COVID 19	28 (30.1)	12 (42.9)	16 (57.1)	

Data are presented as Number (N), percentage and Mean±SD (standard deviation).*chi-squared test and ** independent t-test were used to compare the two groups at baseline. Comorbidity: having one or more of coexisting conditions such as end-stage heart failure, recent cardiac intervention less than 2 months, pulmonary fibrosis or advanced COPD, end-stage kidney disease or liver disease, active tuberculosis or active hepatitis, or Classify stage of COVID 19 according to the Eight National Guidelines for the Treatment of COVID-19 Note: p≤.05, significant

**Outcome measures**

**Effect** **on PO**_2_

Table 2. shows that regardless of the patient's characteristics, the degree of PO_2_ in the two groups was similar at the beginning, but after the treatment it was higher in Phytopaj / ordinary treatment than in the ordinary treatment group (From 91.86 to 93.22 mmHg in the Phytopaj / ordinary treatment group, and from 91.41 to 91.91mmHg in the ordinary treatment group), but they weren’t statistically significant.

After the intervention, within groups evaluation showed that no significant change in mean PO_2_ was found in Phytopaj/ordinary and ordinary treatments (p>0.05).

**Figure 2:**
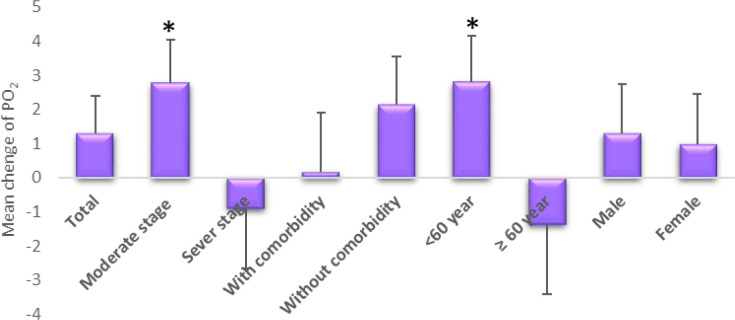
Mean changes and standard deviation (mmHg) of PO_2_ (blood oxygen saturation) in Phytopaj/ ordinary treatment compared to ordinary treatment after 14 days of treatment*, p-value≤0.05

However, Phytopaj / ordinary treatment was significantly effective in the moderate stage of COVID-19 and in participants under 60 years old compared to baseline (p≤0.05). Between groups, the evaluation indicated that after the intervention, Phytopaj/ordinary treatment increased the mean of PO_2_ in the moderate stage of COVID-19 and in participants under 60 years old compared to ordinary treatment (p≤0.05) (Table2 and Figure 2). There was an inverse effect of this treatment in the severe stages of COVID-19 and in the elderly (p>0.05).

**Table 2:** Comparison means of oxygen saturation (PO_2_) at baseline and after 14-day intervention

	Ordinary treatment						Phytopaj / ordinary treatment			Differences between groups (p-value)	
Variables	Baseline		Day14		p-value		Baseline	Day14	p-value	Before Int.	After Int.
Total	91.41(9.18)		91.91(5.92)		0.62		91.86(4.62)	93.22(4.26)	0.06	0.76	0.22
Moderate stage of COVID 19	93(5.44)		92.16(6.04)		0.21		92.92(4.31)	94.92(3.75)	0.01	0.95	0.03
Severe stage of COVID 19	86.5(15.37)		92.16(5.71)		0.21		90(4.69)	90.25(3.47)	0.86	0.39	0.60
Male	90.36(10.65)		91.15(6.41)		0.60		91.11(4.57)	92.44(4.57)	0.23	0.73	0.37
Female	88.56(4.51)		88.5(4.53)		0.89		88.05(4.58)	89.47(4.04)	0.08	0.75	0.52
With comorbidity	92.82(5.75)		92.06(6.44)		0.77		92.17(5.43)	92.23(4.16)	0.39	0.70	0.92
Without comorbidity	90.8(12.79)		91.7(5.23)		0.69		92.29(4.09)	93.85(4.29)	0.10	0.57	0.12
**Age**										
<60 year	90.04(11.96)		90.79(6.22)		0.71		91.86(4.53)	93.58(4.53)	0.03	0.41	0.04
≥60 year	92.72(5.27)		93(5.53)		0.65		91.87(5.35)	91.62(2.38)	0.88	0.69	0.50

Data are presented as Mean±SD (standard deviation). Independent samples t-test was used for statistical analysis between groups and dependent sample t-test was used for statistical analysis within groups. Int=intervention;

Classify according to the Eight National Guidelines for the Treatment of COVID-19

Comorbidity: having one or mor of coexisting conditions such as end-stage heart failure, recent cardiac intervention less than 2 months, pulmonary fibrosis or advanced COPD, end-stage kidney disease or liver disease, active tuberculosis or active hepatitis, or undergoing immunosuppressive treatment

**Effect** **on peripheral blood lymphocytes count (LC)**

Table 3 shows that the mean peripheral blood lymphocyte count was similar at the beginning of the study in the two treatment groups. 

**Figure 3:**
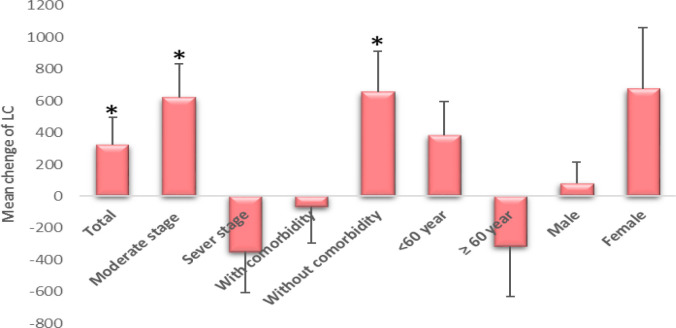
Mean changes and standard deviation (/μl) of LC (peripheral blood lymphocyte count) in Phytopaj/ ordinary treatment compared to ordinary treatment after 14 days of treatment. *, p-value≤ 0.05

Following the intervention, within groups evaluation showed that Phytopaj/ordinary and ordinary treatments were significantly increased the mean peripheral blood lymphocyte count (p≤0.05). However, Phytopaj / ordinary treatment was significantly effective in all characters of participants (p≤0.05) except elderly age. On the other hand, ordinary treatment was significantly effective in the severe stages of COVID-19 and in women. Between groups, the evaluation indicated that after the intervention, Phytopaj/ordinary treatment increased the mean peripheral blood lymphocyte count in total and in the moderate stage of COVID-19 and in participants without comorbidity (p≤0.05) (Table 3 and Figure 3). There was an inverse effect of this treatment in the severe stages of COVID-19 and in the elderly (p>0.05).

**Table 3:** Comparison means of peripheral blood lymphocyte count (LC) at baseline and after 14-day intervention

	**Ordinary treatment**							** Phytopaj / ordinary treatment**					**Differences between groups (p-value)**		
**Variables**	**Baseline**		**Day14**		**p-value**		**Baseline**		**Day14**		**p-value**		**Before Int.**	**After Int.**	
**Total**	1104.28(543.61)		1326.12(719.28)		0.01		1015.90(500.55)		1652.27(921.38)		0.00		0.41	0.05	
**Moderate stage of COVID 19**	1090.81(555.65)		1207.56 (709.47)		0.17		1067.85(508.48)		1830.35(1003.63)		0.00		0.86	0.00	
**Severe stage of COVID 19**	1145.83(525.90)		1691.66(645.90)		0.04		925(488.87)		1340.62(676.56)		0.02		0.26	0.17	
**Male**	1108.48(579.72)		1241.81(512.05)		0.20		951.85(494.67)		1324.07(509.78)		0.00		0.27	0.53	
**Female**	1095.62(478.09)		1500(1023.88)		0.03		1117.64(507.73)		2173.52(1178.46)		0.00		0.89	0.09	
**With comorbidity**	1188.96(608.49)		1425.17(840.17)		0.08		1005.88(548.54)		1358.82(539.74)		0.04		0.31	0.77	
**Without comorbidity**	981.5(417.26)		1182.5(479.65)		0.10		1022.22(478.64)		1837.03(1064.42)		0.00		0.76	0.01	
**Age**														
**<60 year**	1161.66(526.06)		1433.75(568.01)		0.06		1091.66(513.87)		1818.05(916.37) 906.25(488.75)		0.00		0.61	0.07	
**≥60 year**	1049.2(565.13)		1222.8(838.34)		0.14		675(237.54)				0.22		0.08	0.32	

Data are presented as Mean±SD (standard deviation). Independent samples t-test was used for statistical analysis between groups and dependent sample t-test was used for statistical analysis within groups. Int=intervention;

Classify according to the Eight National Guidelines for the Treatment of COVID-19

Comorbidity: having one or mor of coexisting conditions such as end-stage heart failure, recent cardiac intervention less than 2 months, pulmonary fibrosis or advanced COPD, end-stage kidney disease or liver disease, active tuberculosis or active hepatitis, or undergoing immunosuppressive treatment

## Discussion

Despite the problem of COVID-19, there are no effective and accessible treatments available worldwide (Wang et al., 2020; Esmaeilzadeh et al., 2023), and financial sanctions and lost oil revenues have made it more difficult for Iranians to deal with the epidemic and cure patients (Abdollahzadeh et al., 2021; Esmaeilzadeh et al., 2020; Esmaeilzadeh et al., 2023). To combat these problems, our country's traditional and natural resources must be investigated and utilized. The use of medicinal herbs with antiviral activity as supportive treatments or treatment for viral infections has been proven in several studies (Shi et al., 2021; Shi et al., 2021). COVID-19 pathogenesis can be inhibited by medicinal plants by reducing replication of SARS-CoV-2 and its entry into host cells. Among the antiviral medicinal plant species, citrus Spp., Allium sativum, Allium cepa, Nigella sativa, and Mentha piperita are the most effective medicinal plants for the treatment of COVID-19 (Demeke et al., 2021; Sapra et al., 2021). 

In this study, Phytopaj/ ordinary treatment were administered to hospitalized COVID-19 patients in a randomized, single-blind, reference manual-controlled trial. Based on our findings, Phytopaj/ ordinary treatment significantly improved PO_2_ and LC based on the stage of the disease and patient characteristics. Thus, patients with moderate stage and without comorbidity as well as younger ones responded better to treatment. Female patients responded well to the drug as well.

Hydrogen peroxide (H_2_S) is an endogenous mediator and signaling molecule. It has been established that a variety of natural and synthetic molecules are potent donors of H_2_S, and some of them are currently being evaluated in clinical trials for their efficacy as a treatment for diabetes, atherosclerosis, cardiovascular disorders, inflammation, neurodegeneration, cancer, sepsis, asthma and other conditions (Jiang et al., 2018; Polhemus et al., 2015; Wallace and Wang, 2015; Wang, 2012). Biological thiols (such as glutathione (GSH)) react with organic polysulfides, trisulfides, and disulfides to release hydrogen sulfide (H_2_S) (Pluth et al., 2015). The release of H_2_S from disulfides is usually slower than that from trisulfides (Benavides et al., 2007; Liang et al., 2015).

The authors of a recent study found that patients with a favourable outcome of COVID-19 pneumonia had a higher level of H_2_S than those with a severe illness. In addition, serum H_2_S was correlated negatively with interleukin-6 (IL-6) and positively with lymphocyte count (Renieris et al., 2020). 

As a result of this study, decreased H_2_S bioavailability could indicate an increased pro-inflammatory response, so H_2_S donor agents could be administered in order to counteract the severe consequences of COVID-19 infection by restoring H_2_S plasma levels (Renieris et al., 2020). COVID-19 patients suffered from severe lung injuries, respiratory distress, and higher mortality rates resulting from the cytokine storm caused by IL-6 (Gubernatorova et al., 2020). According to another study, slow H_2_S donors reduced the levels of proinflammatory cytokines like TNF-α, IL-1β, and IL-6 in a rat model of lipopolysaccharide-caused lung inflammatory. In addition, H_2_S suppression of IL-8 expression in the lungs and plasma is associated with a significant increase of anti-inflammatory IL-10 levels (Faller et al., 2018). The lungs are infiltrated with neutrophils in COVID-19-associated pulmonary disease. The infiltration of neutrophils in the lungs is inhibited by H_2_S and disulfides (Faller et al., 2018; Liu et al., 2018; Zanardo et al., 2006). Additionally, H_2_S donors facilitate thrombolysis by reducing platelet-leukocyte aggregation, which reduces thrombus stability (Finsterbusch et al., 2018; Grambow et al., 2017). H_2_S also inhibits ROS in neutrophils, improving the prophylactic ability of H_2_S *-donors* (Faller et al., 2018). An animal model of acute lung injury induced by LPS was used to examine the lung protective effects of sulforaphane as a natural H_2_S donor. Researchers found that sulforaphane decreased proinflammatory mediator release and improved mitochondrial function and energy metabolism by activating Nrf2 in cells (Lucarini et al., 2018). By increasing nuclear translocation of Nrf_2_*, *H_2_S *-*donors promote the production of antioxidant genes and protect against oxidative damage (Gojon and Morales, 2020). GYY4137, an H_2_S *-*donating molecule, reduced proinflammatory cytokines like TNF, IL-1, and IL-6 in an LPS-induced pulmonary inflammation model in rats. As well as exhibiting antioxidant properties, it stimulated the activity of antioxidant enzymes such as SOD and catalase in the lung tissues, resulting in a balance between GSH and GSSG in the tissues (Faller et al., 2018). NF-b migration into the nucleus is inhibited by H_2_S donors, which reduces pulmonary vascular inflammation, arterial hypertension, and cytokine production (Faller et al., 2018; Zhang et al., 2019). Mucolytic activity and mucus viscosity are decreased by endogenous H_2_S and low doses of exogenous H_2_S (Bazhanov et al., 2017). Mucins are damaged by disulfide bonds (Costantino et al., 2006). As a result of H_2_S activating ATP-sensitive potassium channels (KATP*)*, bronchodilation is enhanced, while reducing sodium/potassium ATPase activity and calcium-sensitive potassium channels, which promote electrolyte absorption and mucociliary clearance (Pouokam and Althaus, 2016). Angiotensin-converting enzyme 2 (ACE2) and transmembrane protease serine 2 (TMPRSS2) are two host proteins that allow SARS-CoV-2 to enter cells. The authors suggest that H_2_S interferes with ACE2 and TMPRSS, prevents SARS-CoV-2 from infecting host cells, inhibits viral assembly and release, and protects against SARS-CoV-2-induced lung injury by inhibiting immunosuppression and antiinflammatory effects (Citi et al., 2020). *Ferula assa-foetida* gum contains thiophene, disulfides and polysulfide derivatives, among other organic sulfides E/Zsec-butyl propenyl disulfide and E/Z (methylthio)propyl 1propenyl disulfide were identified as the major constituents of *Ferula assa-foetida gum.* It appears that the combination of *Ferula assa-foetida* and tragacanth (phytopaj) had a positive effect on hospitalized COVID-19 patients. As a result of its anti-inflammatory, antiviral, and antioxidant effects, Phytopaj appears to act as an H_2_S donor agent against lung injury caused by SARS-CoV-2.

The essential oil of *Ferula assa-foetida* *are *α-pinene, β-pinene, myrcene and limonene (Dehpour et al., 2009). Animal model studies showed that these agents induced relaxation of isolated ileum (Bagheri et al., 2020). These antispasmodic effect of FAFSEO can support the traditional claim of *Ferula assa-foetida *to relaxing mussels in respiratory diseases. 

This study was limited by its short follow-up duration, its lack of certainty about the cure, and the fact that some patients lost interest and stopped the treatment.

In conclusion, our study found that using phytopaj could be beneficial in treating COVID-19 patients in moderate stage and those without comorbidities and <60 years of old. In comparison to ordinary treatments, it could effectively improve degree of PO_2_ and LC. Through their anti-inflammation, antiviral, and antioxidant properties, phytopaj may function as H_2_S -donor agents against SARS-CoV-2 induced lung injury. However, further studies are necessary to confirm this function of H_2_S. The study also found that phytopaj should not be prescribed to elderly patients or patients with underlying diseases until important insights have been provided. 
